# The hypermorph FtsA* protein has an *in vivo* role in relieving the *Escherichia coli* proto-ring block caused by excess ZapC^+^

**DOI:** 10.1371/journal.pone.0184184

**Published:** 2017-09-06

**Authors:** Cristina Ortiz, Mercedes Casanova, Pilar Palacios, Miguel Vicente

**Affiliations:** Centro Nacional de Biotecnología- Consejo Superior de Investigaciones Científicas, Madrid, Spain; Centre National de la Recherche Scientifique, Aix-Marseille Université, FRANCE

## Abstract

Assembly of the proto-ring, formed by the essential FtsZ, FtsA and ZipA proteins, and its progression into a divisome, are essential events for *Escherichia coli* division. ZapC is a cytoplasmic protein that belongs to a group of non-essential components that assist FtsZ during proto-ring assembly. Any overproduction of these proteins leads to faulty FtsZ-rings, resulting in a cell division block. We show that ZapC overproduction can be counteracted by an excess of the ZipA-independent hypermorph FtsA* mutant, but not by similar amounts of wild type FtsA^+^. An excess of FtsA^+^ allowed regular spacing of the ZapC-blocked FtsZ-rings, but failed to promote recruitment of the late-assembling proteins FtsQ, FtsK and FtsN and therefore, to activate constriction. In contrast, overproduction of FtsA*, besides allowing correct FtsZ-ring localization at midcell, restored the ability of FtsQ, FtsK and FtsN to be incorporated into active divisomes.

## Introduction

The initial steps in the division of most bacteria involve polymerization of the cytoplasmic bacterial tubulin homologue FtsZ to form a constricting ring, the FtsZ-ring [1, 2]. In *Escherichia coli*, the proto-ring (FtsZ, FtsA and ZipA) serves as a scaffold for the recruitment of at least 13 essential proteins [3]. FtsZ is a cytoplasmic protein that attaches to the cytoplasmic membrane through the FtsA and ZipA proteins [4, 5], whose assembly and localization depend solely on FtsZ; together, these three proteins form the proto-ring structure [6]. In addition to these essential divisome components, a group of non-essential proteins known as the FtsZ-associated proteins (ZapA, B, C, D and ZapE) assist FtsZ during proto-ring assembly [7], acting as stabilizers of the FtsZ-ring [8].

The widely conserved FtsA binds the membrane through its C-terminal amphipathic helix [9–11, whereas ZipA, a protein restricted to the gammaproteobacteria, is integrated into the membrane through its N-terminal transmembrane domain [5, 12]. These two proteins interact with FtsZ through the FtsZ central hub located at its C-terminal domain [13, 14, 15, 16]. Overproduction or depletion of FtsA or ZipA results in a block in FtsZ-ring assembly, thus halting cell division progress [5, 17, 18]. *In vivo*, a point mutation in the FtsA protein (R286W), FtsA* can bypass the ZipA requirement in cell division [19] by suppressing the blockade caused by lack of ZipA or excess of FtsZ. In the presence of FtsA*, mean cell length is nevertheless shorter than normal, and the FtsZ-ring is more stable than in the presence of FtsA^+^ [20]. Overproduction of FtsA* is less damaging for cell division than that of FtsA^+^ [20].

Although Zap proteins have redundant roles during cell division by stabilizing the FtsZ-ring, they share no structural similarities [8]. Individual Zap proteins are not essential for cell division progression, as shown by the behavior of mutants in which only one is defective [8, 21]; cell division defects nonetheless occur when more than one is mutated [22]. *In vivo*, ZapA [23], ZapB [24], ZapC [21] and ZapD [8] co-localization is FtsZ-dependent and *in vitro* they interact directly with the FtsZ polymers to induce bundling, as observed by electron microscopy [8, 21, 23, 24]. Although none could be crystallized in complex with FtsZ, the ZapA, ZapB, ZapC and ZapD crystal structures have been solved [24–27]. Biochemical experiments determined the different interacting sites between these proteins and FtsZ [28]. Although several FtsZ interaction sites have been identified in ZapC, it remains unclear how the Zap proteins regulate FtsZ assembly [26]. ZapC is a substrate for the ClpXP protease complex, which influences the FtsZ-ring dynamics by altering the pool of FtsZ subunits in the cell [29]. *In vivo* data confirm direct ZapC interaction with FtsZ, as its overproduction blocks progression of cell division, and aberrant FtsZ-ring structures can be observed dispersed along the filamented cells [21].

We analyzed *in vivo* whether the impairement of the FtsZ-ring function observed in the presence of a ZapC excess can be restored leading to an active ring dynamics by overproduction of other cell division proteins. Since Zap proteins localize with FtsZ at the midcell during the early stage of FtsZ recruitment [8, 21, 24, 30], we focused on the effect of additional proto-ring components, FtsA and ZipA. The hypermorph FtsA* mutant was overproduced to determine whether FtsA* can bypass the lethal blockade caused by ZapC on the FtsZ-ring. We observed that in the presence of an excess of ZapC, the excess of FtsA^+^ or FtsA* had different effects on the FtsZ-rings correlating with their different ability to recruit the late assembly proteins FtsQ, FtsK and FtsN needed for the production of an active divisome.

## Materials and methods

### *Escherichia coli* strains and growth conditions

Strain CH59 [21] (S1 Table), a Δ*zapC E*. *coli*, was used for *in vivo* studies of *zapC*_*his*_, *zipA*^*+*^, *ftsA*^*+*^, ftsA* overproduction and double overproduction of *zapC*_*his*_ and *zipA*^*+*^, *zapC*_*his*_ and *ftsA*^*+*^, *zapC*_*his*_ and ftsA*. *E*. *coli* CH59 cells transformed with pMPV1, pASV003, pPNV40, pPZV33 and double transformation of both pMPV1 and pASV003, pMPV1 and pPNV40, pMPV1 and pPZV33 (S2 Table) were inoculated in Luria–Bertani (LB) broth supplemented with ampicillin (100 μg/ml) and chloramphenicol (50 μg/ml), based on the resistance of each plasmid, and glucose 0.2% to repress gene expression.

Cells were cultured (overnight, 37°C) and then diluted (1:50) in fresh pre-warmed LB medium. Optical density at 600 nm (OD600) was measured periodically with a CO8000 Cell Density Meter (WPA biowave) and maintained below 0.3 with pre-warmed medium a shaking water bath with aeration, to attain exponential balanced growth for at least 4 mass doublings [52]. Cells were then filtered and transferred to medium with 0.2% arabinose or 0.5 mM IPTG (180 min), depending on the promoter of each plasmid. As negative control, we used the corresponding empty expression vector.

### Cell parameter measurements

Samples were removed from cultures at 30-min intervals for 180 min and fixed in 0.75% formaldehyde. The number of particles per volume was determined using a Beckman Coulter Multisizer 3 multi-channel analyzer equipped with a 30 μm-diameter orifice. The mean length of 100 cells was measured at time 0, 60 and 120 min. Fixed cells were analyzed with ImageJ software (NIH) and processed with Excell software.

### Western blotting

Cells were harvested by centrifugation, and lysed by suspension in SDS-PAGE (sodium dodecyl sulphate-polyacrylamide gel electrophoresis) sample loading buffer [31] to the equivalent of 0.3 OD600 units/ml, and heated (5 min, 95°C). Proteins were resolved by 12% SDS-PAGE [32] and analyzed by Western blotting [33]. Membranes with transferred proteins were incubated (1 h) with primary rabbit antibody MCV2 (1:20,000 dilution) specific for FtsZ protein, MVC3 (1:400) specific for FtsA^+^ and FtsA* proteins, and MVC1 (1:10,000) specific for ZipA^+^ protein, followed by peroxidase-coupled protein A (1:3000, Bio Rad; 1 h) as secondary antibody (S3 Table).

Overproduced ZapC protein was detected using anti-histidine monoclonal antibody clone His-1 (peroxidase conjugate, Sigma Aldrich A7058; 1:10,000, 30 min) (S3 Table), membranes were developed with the BM chemiluminescence blotting substrate (POD; Roche), and luminescence signals were developed with the ChemDoc XRS+ Imaging system or Kodak Biomax XAR film.

### Fluorescent immunomicroscopy

Cell samples for immunofluorescence microscopy were obtained and processed as described [34]. Cell samples were fixed with methanol/acetic acid (4:1) and adhered to poly-L-lysine pre-treated coverslips, and permeabilized with lysozyme (100 μg/ml, 2 min). Non-specific binding sites were first blocked by incubating cells in 2% bovine albumin (BSA, Serva) in PBS (20 min), followed by incubation (overnight, 4°C) with purified antibodies diluted in blocking solution. FtsZ, ZipA^+^, FtsA^+^, FtsA*, FtsQ, FtsK and FtsN proteins were detected with antibodies MVC2, MVC1, MVC3, MVC9, MVC6, MVG1 (S3 Table). Unbound primary antibodies were removed by extensive washing, followed by incubation with secondary antibody Alexa 594-conjugated anti-rabbit antibody (Invitrogen A-11037) (S3 Table) to detect the proteins (red signal). The nucleoids were stained with DAPI (25μg/ml) (Sigma D9542). Cover slips were mounted in Vectashield medium (Vector Laboratories) and sealed.

Cells were imaged with a Hamamatsu 3CCD Digital Camera C7780 coupled to a BX61 Olympus fluorescence microscope, equipped with an 100x immersion oil lens. The filters used to detect the red signal (proteins) or the blue signal (nucleoids) were U-MWTY2 and U-MNU2 respectively. The images were captured and deconvolved with SimplePCI imaging software. Intensity levels and image overlay were adjusted using Adobe Photoshop CS3.

## Results

### Neither high FtsA^+^ nor ZipA^+^ levels counteract the blockade in cell division caused by *zapC*^*+*^ overexpression

**Fig 1:**
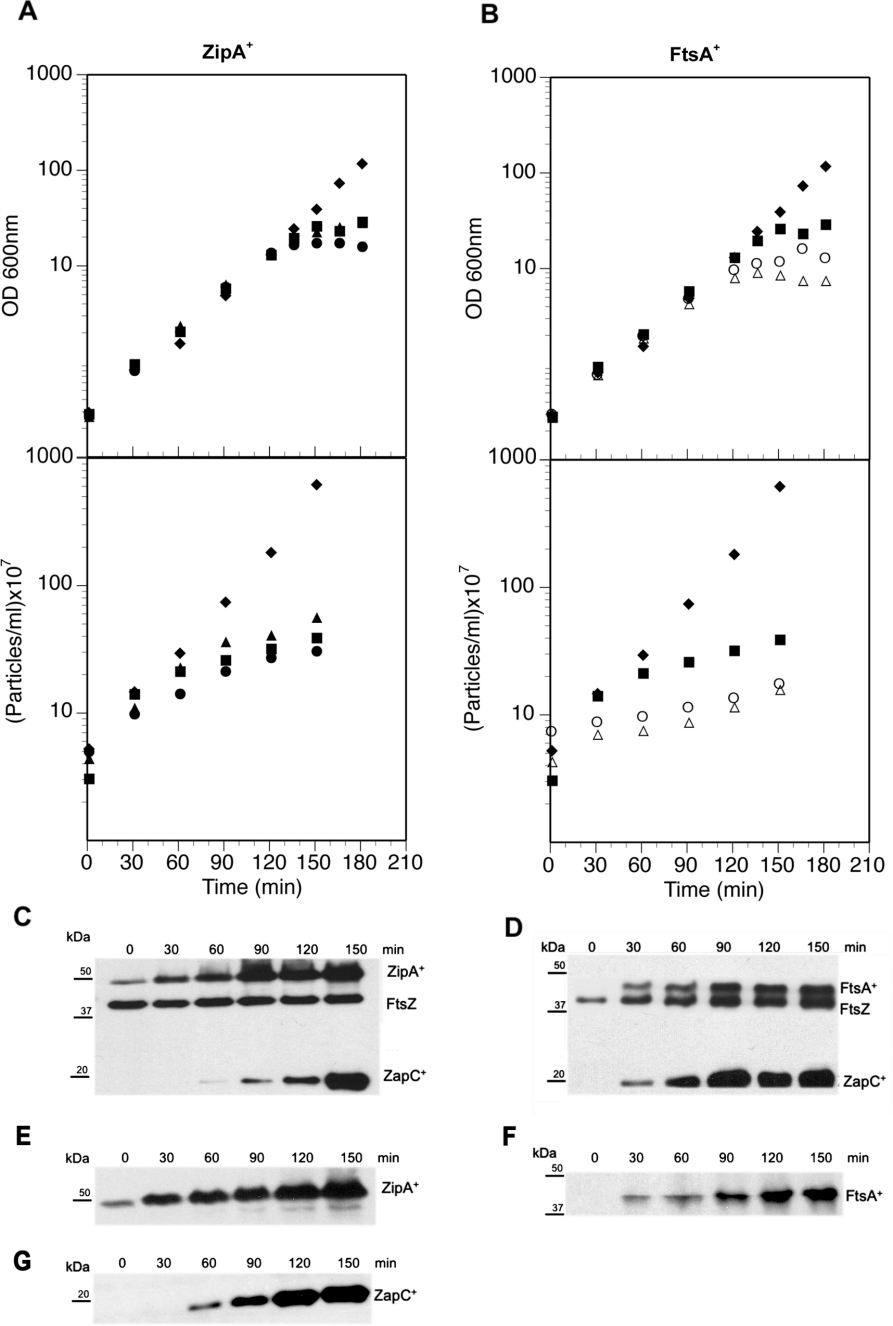
Effect of *zipA*^*+*^, *ftsA*^*+*^ and *zapC*^*+*^ overexpression on cell growth and division. **A.** Cultures of several CH59 (*ΔzapC*) transformed with different plasmids were grown in glucose-containing LB medium. Strain VIP2004 bears pBAD33 (empty vector; ◆), VIP2003 bears pMPV1 (*zapC*^*+*^; ■), VIP2011 bears pASV003 (*zipA*^*+*^; ▲), VIP2012 bears pMPV1 and pASV003 (*zapC*^*+*^*-zipA*^*+*^; ●). At time 0, 0.2% arabinose was added to induce the expression of *zapC*^*+*^ (pMPV1), *zipA*^*+*^ (pASV003). Optical density (600 nm) and particle increase were measured at the times indicated (plotted values corrected for dilutions). **B.** Cultures of several CH59 (*ΔzapC*) were transformed with different plasmids. Strains VIP2004 (*ΔzapC*) bearing pBAD33 (empty vector; ◆), VIP2003 bearing pMPV1 (*zapC*^*+*^; ■), VIP2009 bearing pPNV40 (*ftsA*^*+*^; *Δ*), VIP2010 bearing pMPV1 and pPNV40 (*zapC*^*+*^*-ftsA*^*+*^;O) were treated as in A, in addition 0.5 mM IPTG was added to induce the expression of *ftsA*^+^ (pPNV40). **C.** Samples corresponding to the culture overexpressing together *zipA*^*+*^ and *zapC*^*+*^ were withdrawn at indicated times post-induction and analyzed by Western blot using anti-ZipA and -FtsZ antibodies to detect the induced levels of ZipA^+^ and intracellular FtsZ levels. **D.** Samples corresponding to the culture overexpressing together *ftsA*^*+*^ and *zapC*^+^ were treated as in C, and Western blot was developed using anti-FtsA^+^, -FtsZ and -histidine antibodies to detect FtsA^+^, FtsZ and ZapC^+^ levels. **E-G.** Samples corresponding to cultures in which *zipA*^*+*^ (E), *ftsA*^*+*^ (F) and *zapC*^*+*^ (G) were overproduced separately, were treated as in C.

Overproduction of ZapC^+^ results in a block of cell growth and division, probably caused by loss of the dynamic behaviour of the FtsZ polymers in the FtsZ-ring [21]. Since the relative amount of proto-ring components can modify FtsZ polymer dynamics [35], we examined the effect of overproducing ZapC^+^, together with ZipA^+^ or FtsA^+^, on *E*. *coli* cell growth and division. We measured the increase in optical density and particle numbers in cultures of strains bearing suitable compatible plasmids from which ZipA^+^ or FtsA^+^ as well as ZapC^+^ were overproduced (Fig 1A and 1B; see Methods).

The *E*. *coli ΔzapC* strain CH59 was transformed with plasmids expressing *zapC*^*+*^, *zipA*^+^ and *ftsA*^+^ (S1 and S2 Tables). Results showed that the mass of all cultures increased initially at a constant rate (Fig 1A). At 150 min post-induction, the rate of mass increase in cultures expressing *zapC*^*+*^ or *zipA*^*+*^ alone or *zapC*^*+*^*-zipA*^*+*^ slowed (see Methods), whereas the mass increase of the culture with empty vector was unaffected. We tested whether high ZapC^+^, ZipA^+^ and ZapC^+^-ZipA^+^ levels also affect particle increase, and found that, after two mass doublings (60 min; Fig 1A), overproduction of any of these proteins clearly curbed the rate of particle increase. Given that the correct FtsA:FtsZ ratio has an important effect on division [17, 18], we tested the possibility that overproduction of FtsA^+^, the other component of the proto-ring, would reverse the block in cell division (Fig 1B). Mass and particle increase slowed, as in ZipA^+^-overproducing cultures. Moreover, in contrast to the effect of *zipA*^*+*^ and *zapC*^*+*^ overexpression, cultures overexpressing *ftsA*^*+*^ and *zapC*^*+*^-*ftsA*^*+*^ showed a acute halt in cell division after one mass doubling (30 min) (Fig 1B). We used Western blot analysis to rule out an effect of ZapC^+^, ZipA^+^ or FtsA^+^ overproduction on the amount of FtsZ; FtsZ levels were unaffected by ZipA^+^ overproduction and only slightly altered when FtsA^+^ was overproduced together with ZapC^+^ (Fig 1C and 1D). When the overproduced proteins were analysed separately, we found no significant difference in the levels of each individual protein in comparison to the cultures overexpressing both at the same time (Fig 1E–1G). We conclude that neither of the other two proto-ring components counteracts the cell division blockade caused by excess ZapC^+^.

### FtsZ localizes differently in strains overproducing ZapC^+^, FtsA^+^ or ZipA^+^

**Fig 2:**
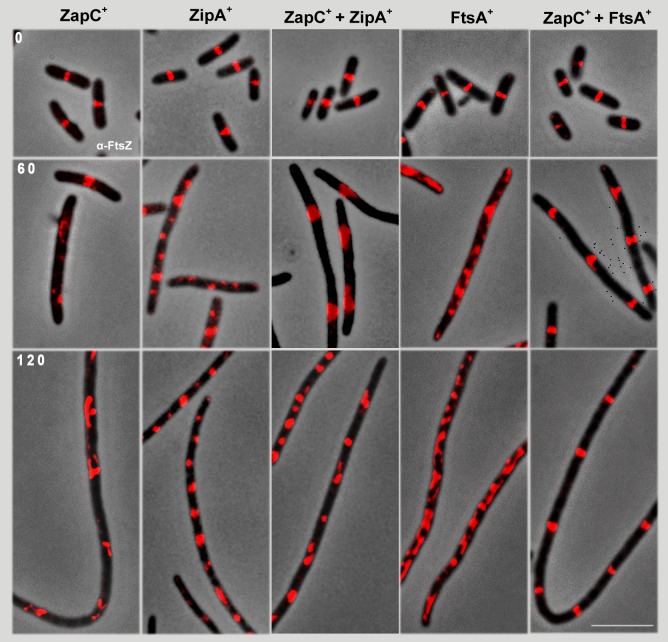
Effect of ZapC^+^, ZipA^+^ and FtsA^+^ overproduction on FtsZ-ring localization. Samples from the cultures in Fig 1A and 1B were withdrawn at indicated times. FtsZ protein was visualized in all cultures using anti-FtsZ and Alexa 594-conjugated anti-rabbit antibody (red). Bar: 5 μm.

We studied FtsZ-ring assembly and localization in conditions in which ZapC^+^, ZipA^+^, FtsA^+^ and their combinations were overproduced. Samples from cultures overexpressing *zapC*^*+*^, *zipA*^*+*^ or *ftsA*^*+*^ and their combinations were withdrawn at different times post-induction and used to immunolocalize FtsZ (Fig 2). At time 0, all strains bearing *zapC*^*+*^, *zipA*^*+*^, *ftsA*^*+*^, alone or in combination, showed the FtsZ protein located at the midcell, forming the FtsZ-ring as anticipated (Fig 2). After 60 min of ZapC^+^ overproduction, FtsZ was not organized into discrete rings in all cells; after 120 min, it became further disorganized. Overexpression of *zipA*^*+*^ alone or with *zapC*, allowed FtsZ to localize at discrete intervals, forming irregular accumulations. Overexpression of *ftsA*^*+*^ alone did not result in regular FtsZ distribution. The FtsZ fluorescence signal in filamentous cells co-overproducing ZapC^+^ and FtsA^+^ assembled into sharp rings regularly spaced at discrete positions. As growth continued, inactive rings assembled at wider intervals along the filament, separated on average by 4.63 μm at 60 min or 4.59 μm at 120. This distance is approximately 150% the mean length of dividing cells at time 0 (3.08 μm; Fig 2, and Methods), which indicated that potential division sites are spaced at greater distances that in normal division conditions. These observations were confirmed by DAPI staining (S1 Fig) in which the nucleoids appear well segregated but not all the potential septation sites contain an FtsZ-ring.

### The late assembly proteins FtsQ, FtsK and FtsN do not gather as rings in cells that simultaneously overexpress *zapC*^*+*^ and *ftsA*^*+*^

**Fig 3:**
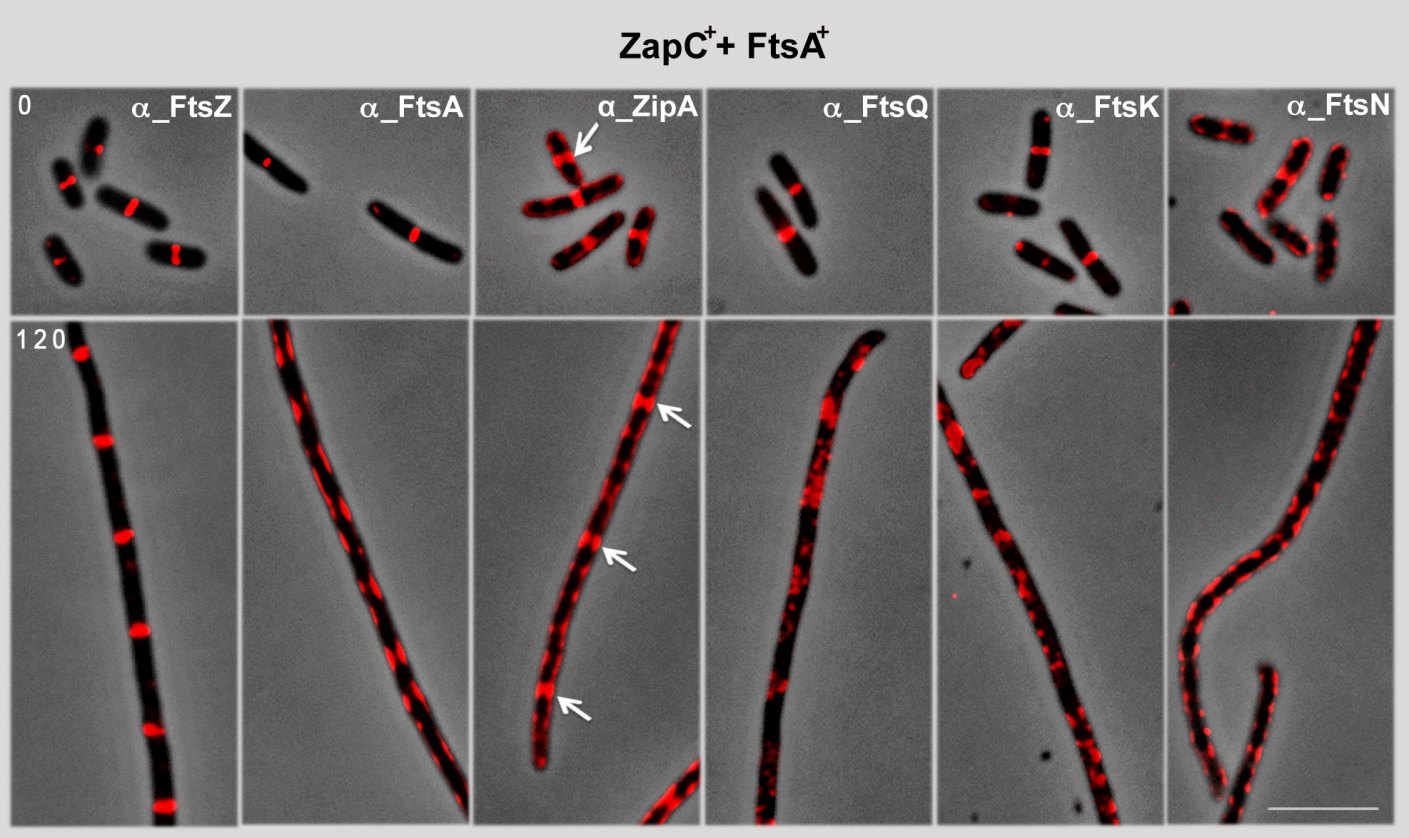
Effect of simultaneous ZapC^+^ and FtsA^+^ overproduction on recruitment of late assembly divisome components. Samples were withdrawn at times indicated and FtsZ, FtsA, ZipA, FtsQ, FtsK and FtsN proteins were visualized using specific antibodies (S3 Table) and Alexa 594-conjugated anti-rabbit antibody (red). White arrows indicate ZipA localization as rings. Bar: 5 μm.

FtsZ-rings in the *zapC*^*+*^-*ftsA*^*+*^-overexpressing cells, even when regularly localized, failed to become active in division. We thus tested whether this was due to proto-ring inability to recruit the late assembly cell division proteins FtsQ, FtsK and FtsN together with FtsZ to form active divisomes. The FtsA^+^ 1C subdomain has a central role in recruiting other divisome proteins such as FtsN [36]. If FtsA^+^ were able to recruit additional divisome proteins such as FtsN to the FtsZ-ring, cell division could progress to septation. Immunolocalization of each cell division protein was therefore analyzed in cells simultaneously overexpressing *zapC*^*+*^ and *ftsA*^*+*^ (Fig 3). At time 0, all division proteins tested, including transmembrane ZipA^+^, FtsQ^+^, FtsK^+^ and FtsN^+^, were located at the midcell. In contrast, at 120 min these proteins were dispersed along the length of the filament, with the exception of FtsZ and ZipA, which localized as regularly spaced rings (Fig 3).

These experiments also showed that, in the conditions in which FtsA^+^ is overproduced with ZapC^+^, FtsA^+^ appears to be completely delocalized, which might explain its failure to recruit the late-assembling divisome protein FtsN. Divisome stability is not guaranteed until FtsN is recruited [36, 16]. Daley *et al*. [37] recently showed that early partial assembly of FtsN in the proto-ring is a requisite for the initiation of constriction [38–40]. Although the FtsZ-ring might be positioned at the potential cell division sites when ZapC^+^ and FtsA^+^ are co-overproduced, these rings are defective due to their failure to incorporate late-assembling proteins and are thus unable to progress to division.

### Overproduction of the hypermorph FtsA* allows FtsZ-ring progression when ZapC^+^ is overproduced

The hypermorph FtsA* bypasses the *in vivo* requirement for ZipA, suppresses the toxicity caused by perturbing the FtsZ:FtsA ratio, and interacts more strongly with FtsZ than does wild type FtsA^+^; FtsA* is sufficient to maintain the FtsZ-ring in the absence of other divisome components such as ZipA or FtsK [19]. We tested whether these FtsA* properties could compensate the cell division block caused by ZapC^+^ overproduction.

**Fig 4:**
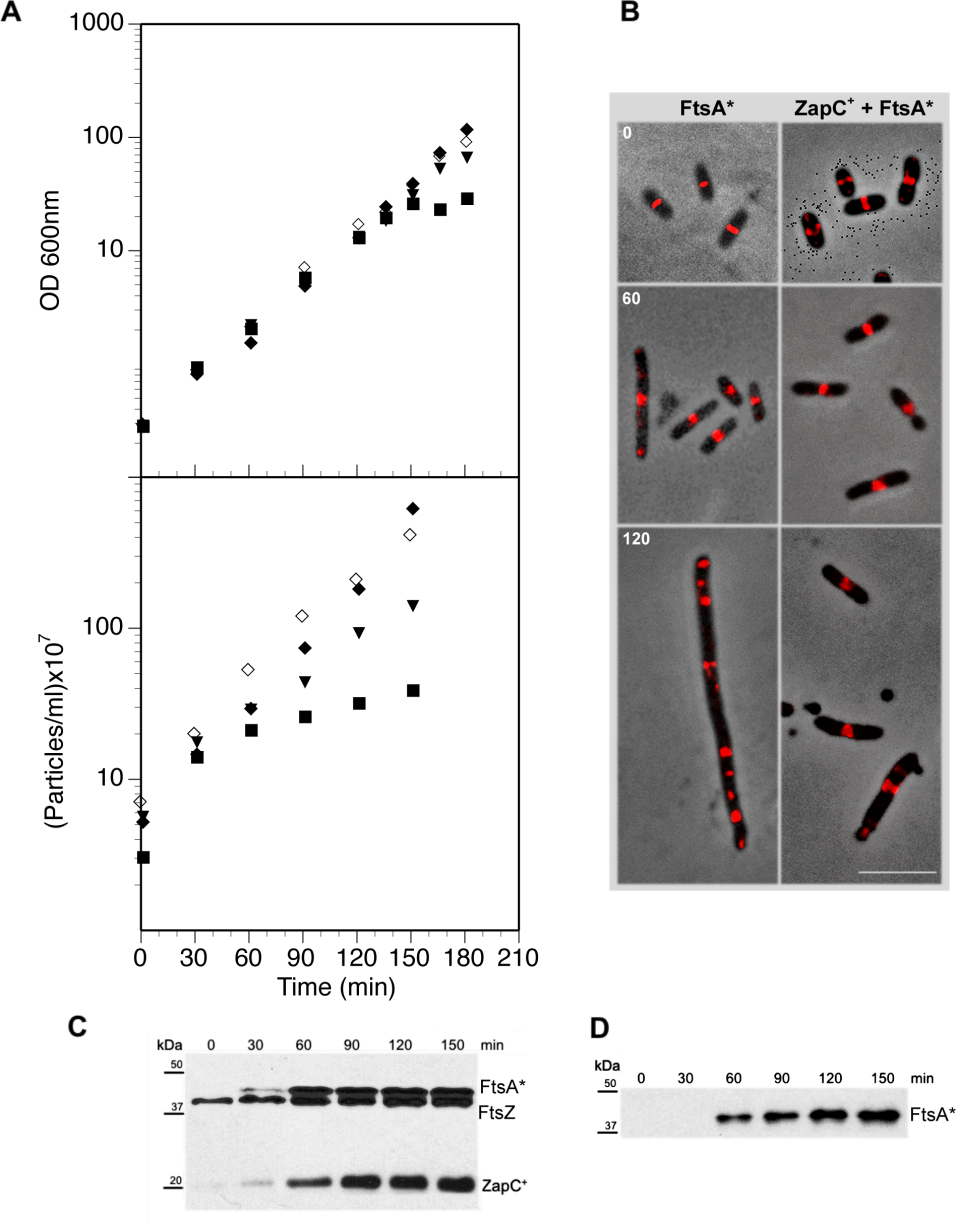
Effect of ftsA* and *zapC*^*+*^ overexpression on growth, division and localization of the FtsZ-ring. **A.** Cultures from strains VIP2004 (*ΔzapC*) bearing pBAD33 (empty vector; ◆), VIP2003 bearing pMPV1 (*zapC*^*+*^; ■), VIP2007 bearing pPZV33 (ftsA*; ▼), and VIP2008 bearing pMPV1 and pPZV33 (*zapC*^*+*^-ftsA*; ◊) were grown in glucose-containing medium, and induced at time 0 with 0.2% arabinose cultures expressing *zapC*^+^ or 0.5 mM IPTG cultures expressing ftsA*. OD600 and particle increase were measured at indicated times. **B.** Samples from cultures were withdrawn at the times indicated, and FtsZ was visualized using anti-FtsZ and Alexa 594-conjugated anti-rabbit antibody (red signal). Bar: 5 μm. **C.** Samples from the cultures overproducing simultaneously FtsA* and ZapC^+^ were withdrawn at the times indicated and the Western blot was developed using anti-FtsA^+^ and anti-FtsZ antibodies to detect intracellular levels of the induced FtsA* and the FtsZ proteins respectively. **D.** Samples from the culture overproducing FtsA* alone were treated as in C.

We transformed the *E*. *coli ΔzapC* CH59 strain with plasmids that expressed *zapC*^*+*^, ftsA* or *zapC*^*+*^ and ftsA* simultaneously (S1 Table), to overproduce the gene products individually or in combination. Optical density of these cultures increased at a constant rate (Fig 4A), with the exception of the *zapC*^*+*^-overexpressing culture, which ceased growth after 150 min, as predicted (Fig 1). Although cells expressing ftsA* from a multicopy plasmid pZA*Q divide normally [19], we nonetheless observed a lower particle increase rate when FtsA* alone was overproduced (Fig 4A). The particle doubling rate was similar to the *ΔzapC* control culture when FtsA* was overproduced with ZapC^+^. Western blot analysis showed that the levels of the overproduced proteins, FtsZ, FtsA* or ZapC did not change markedly, with the exception of FtsZ levels which slightly increases over the induction time, when these proteins were overproduced together relative to the levels observed in the overproduction of each one in isolation (Fig 4C and 4D). The fact that FtsZ levels slightly increased could be due to the interaction with the overproduced FtsA*. It has been reported that the interaction of FtsA* with FtsZ is stronger than with FtsA [20], therefore the FtsZ-rings would have a longer half-life. These results indicate that simultaneous FtsA* and ZapC overproduction allowed cell growth and division at rates similar to the *ΔzapC* control strain, which suggests that FtsA* overproduction counteracts the blocking effect of large amounts of ZapC^+^.

In strains containing ftsA* alone or together with *zapC*^*+*^, the FtsZ protein localized to the midcell before induction, probably forming FtsZ-rings (Fig 4B). When FtsA* was overproduced, the FtsZ fluorescence signal localized initially at discrete rings during the first 60 min, but was delocalized at longer times (120 min). Cells that express ftsA* are shorter than wild type, because high FtsA* levels accelerate FtsZ-ring assembly [20]. At 120 min post-induction, however, the FtsA*-overproducing *ΔzapC* cells were long filaments in which FtsZ was completely disorganized. As predicted from results in optical density and particle increase experiments, the FtsZ-ring was localized at midcell in FtsA*- and ZapC^+^-co-overproducing cells, whose length was similar to that of cells with a complete functional FtsZ-ring. As longer ZapC^+^ and FtsA* induction times also produced mini-cells (Fig 4B, right bottom), we reasoned that large amounts of these proteins might block access of Min and ClpXP, the two main mechanisms that depolymerize and degrade FtsZ (see Discussion). We consider that high FtsA* levels counteract the effects of large amounts of ZapC^+^ on FtsZ, and allow it to assemble into functional rings that can drive constrictions at potential septation sites.

**Fig 5:**
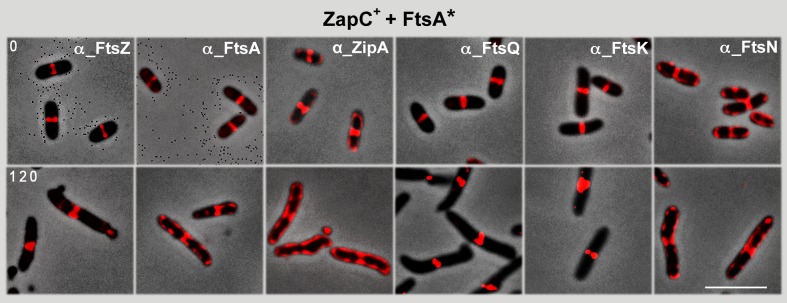
Effect of ZapC^+^ and FtsA* co-overproduction on recruitment of late-assembly divisome components. Samples were withdrawn at times indicated and FtsZ, FtsA, ZipA, FtsQ, FtsK and FtsN proteins were visualized using specific antibodies (S3 Table) and Alexa 594-conjugated anti-rabbit antibody (red). Bar: 5 μm.

We showed that the rings produced by a ZapC^+^ and FtsA^+^ together could not recruit late-assembling proteins and were therefore inactive (Fig 3). To test whether ZapC^+^ blocked FtsZ-rings produced in the presence of excess FtsA* can recover the ability to recruit late divisome proteins, as would be predicted if they are active in division, we immunolocalized FtsQ, FtsK and FtsN in conditions of FtsA* and ZapC^+^ overproduction. At difference from the FtsA^+^ effect, overproduction of the hypermorph FtsA* allowed recruitment of all these late-assembling proteins to the septation site (Fig 5). We propose that, as a result of the high FtsA* levels, the blockade caused by ZapC^+^ overproduction is released and cell division takes place.

## Discussion

FtsZ is currently regarded as the guiding element in divisome assembly [7]. Our findings suggest that divisomes can adopt different topological and functional states depending on the nature and balance of their components. An excess of ZapC^+^, one of the proteins that promotes FtsZ bundling, results in failure of FtsZ to assemble into rings, becoming dispersed along the length of the non-dividing filaments (Fig 2).

We analyzed whether inhibition of cell division caused by ZapC^+^ overproduction could be released by induction of either of the other two proto-ring components, FtsA or ZipA. FtsA^+^ or ZipA^+^ overproduction alone blocks constriction, as it affects the normal FtsZ:FtsA:ZipA ratio [17, 18, 35]. Neither FtsA^+^ nor ZipA^+^ is sufficient to allow FtsZ to produce active rings in the presence of excess ZapC^+^ (Fig 1). Whereas ZipA^+^ is also unable to direct FtsZ to potential septation sites in these conditions, FtsA^+^ overproduction helps to place FtsZ at regularly spaced intervals along the filaments, where it assembles as rings that are nevertheless inactive in septation (Fig 2). It is noteworthy that these abortive rings are regularly separated by a distance 1.5 times the mean length of a dividing cell growing in similar conditions (Fig 2, time 0).

Overproduction of FtsA*, a hypermorph able to bypass most ZipA functions, not only can direct FtsZ to regularly spaced locations but is also able to assemble it into active divisomes (Fig 4). Although FtsZ migrates to the poles before septation is completed [37], polar division is prevented in wild type cells because FtsZ polymerization is normally blocked by the inhibitory effect of MinC [41]. FtsA* can nevertheless relieve the MinC inhibitory effect, which allows division at the cell poles to produce minicells [19]. We found that FtsA* also induces minicell production at the poles of cells in which the FtsZ-ring is blocked by ZapC^+^ overproduction (Fig 4). Interaction of FtsA, FtsA*, ZipA, ZapC and MinC with FtsZ can be established through the FtsZ central hub [7], which is also the region recognized by the ClpXP protease complex to degrade FtsZ [42]. Our results suggest that in the poles of the cells with excess ZapC^+^ and FtsA*, competition of the proteins that interact with the central hub is probably displaced towards the protective role of ZipA rather than the inhibitory or degradative activities of MinC or ClpX, respectively [43]. An excess of ZapC^+^ could further stabilize the FtsZ-ring by stimulating the association of FtsZ protofilaments and by suppressing the GTPase activity [21]. On the other hand, FtsA* would stimulate disassembly of FtsZ [44]. Thus, a balance between the stabilization of the FtsZ-ring by ZapC^+^ and its disassembly by FtsA* could modify the activity of the FtsZ-ring. We propose that FtsZ is accompanied to the poles by a sufficient amount of the other divisome components to allow productive septation. The dynamics of the interaction strength and the dissociation kinetics of several divisome components with the FtsZ central hub needs to be studied in further detail to complete the functional description of the activity of the FtsZ-ring in bacterial division.

*In vitro* studies suggest that even if ZipA and FtsA have a role in anchoring FtsZ to the membrane, they work differently and might have distinct effects on FtsZ-ring dynamics [45]. Whereas FtsA promotes dynamic behavior of FtsZ polymers, ZipA provides a more stable, less dynamic anchor [45]. Our results on the cellular localization of the ZapC-stabilized FtsZ in the presence of excess ZipA^+^ or FtsA^+^ coincide with this view. At high ZapC^+^ and FtsA^+^ levels, the ZipA protein is distributed along the cell membrane and at some potential cell division sites, but does not activate the ZapC-blocked FtsZ-rings (Fig 3). Excess ZipA has a severe effect on the structure of the cytoplasmic membrane, forming abnormal invaginations [46]. These malformations might also contribute to FtsZ misplacement, perhaps by interfering with the Min septum site selection mechanism. No such effect on the membrane is reported when FtsA^+^ is overproduced. The FtsA^+^ excess might allow FtsZ polymers to be sufficiently dynamic to be sensitive to the Min system, but insufficient to remodel the correctly located rings to allow their contractile behavior and progression to productive divisomes (Fig 2). FtsA^+^ polymers, as observed by transmission electron microscopy, adopt a mini-ring structure with no 1C domain free to interact with other proteins; in similar conditions, the FtsA* protein assembles into arcs in which at least one 1C domain is available for heterologous interactions [47, 48]. The availability of the free 1C domain for heterologous interactions in FtsA* polymers would have a greater effect on FtsZ-ring dynamics to promote additional interactions, even when stabilized by ZapC^+^ (Fig 4). This would allow their correct placement by the Min system and production of a contractile structure following FtsZ interaction with other divisome components.

A remarkable situation occurs at the cell poles when both FtsA* and ZapC^+^ are overproduced (Fig 4). In normal cells, the Min system prevents formation of productive FtsZ-rings at the poles [49, 50]. A ZapB or ZapC excess can activate productive divisomes at the poles. This implies that once the septum is closing at midcell, Zap proteins migrate to the poles, carrying sufficient divisome elements with them to activate septation under some conditions [51, 30]. For example, once at the pole, the excess FtsA*, but not FtsA^+^, may trigger FtsZ septation activity by lowering central hub interaction with the FtsZ negative regulators MinC and ClpX, and favoring interactions with activators such as FtsA* itself and the Zap proteins.

## Supporting information

S1 Table *Escherichia coli* strains used in this study. (DOCX)

S2 Table Plasmids used in this study. (DOCX)

S3 Table Antibodies used in this study. (DOCX)

S1 Fig Localization of FtsZ and nucleoids in cells overexpressing *zapC*^+^ and *ftsA*^+^ simultaneously. Samples from the cultures overexpressing together *zapC*^+^ and *ftsA*^+^ were withdrawn at indicated times. Merged images show FtsZ protein visualized using anti-FtsZ and Alexa 594-conjugated anti-rabbit antibody (red signal) and nucleoids visualized using DAPI staining (blue signal). Bar: 5 μm.
(TIF)
