# Supplementary material for: The hypermorph FtsA* protein has an in vivo role in relieving the Escherichia coli proto-ring block caused by excess ZapC+
Source: PLoS One. 2017 Sep 6;12(9):e0184184. doi: 10.1371/journal.pone.0184184 (PMC5587298; doi:10.1371/journal.pone.0184184)
Supplement: S1 Table — (DOCX) [file pone.0184184.s001.docx]

**S1 Table. *E. coli* strains used in this study.**

| **Strain** | **Relevant characteristics** | **Source or reference** |
| --- | --- | --- |
| CH59 | TB28 *zapC<>frt* | (21) |
| TB28 | MG1655 *lacIZYA<>frt* | (21) |
| VIP2003 | CH59 bearing the plasmid pMPV1 | This work |
| VIP2004 | CH59 bearing the plasmid pBAD33 | This work |
| VIP2007 | CH59 bearing the plasmid pPZV33 | This work |
| VIP2008 | CH59 bearing the plasmids pMPV1 and pPZV33 | This work |
| VIP2009 | CH59 bearing the plasmid pPNV40 | This work |
| VIP2010 | CH59 bearing the plasmids pMPV1 and pPNV40 | This work |
| VIP2011 | CH59 bearing the plasmid pASV003 | This work |
| VIP2012 | CH59 bearingt the plasmids pMPV1 and pASV003 | This work |
