# Supplementary material for: The hypermorph FtsA* protein has an in vivo role in relieving the Escherichia coli proto-ring block caused by excess ZapC+
Source: PLoS One. 2017 Sep 6;12(9):e0184184. doi: 10.1371/journal.pone.0184184 (PMC5587298; doi:10.1371/journal.pone.0184184)
Supplement: S2 Table — (DOCX) [file pone.0184184.s002.docx]

**S2 Table. Plasmids used in this study.**

| **Plasmid** | **Relevant characteristics and markers** | **Source or reference** |
| --- | --- | --- |
| pASV003 | pBAD22_zipA_his_, Amp_r_ | Laboratory collection |
| pBAD22 | Cloning vector, contains P_BAD_ promoter, Amp_r_ | Guzman et al. 1995 |
| pBAD33 | Cloning vector, contains P_BAD_ promoter, Cm_r_ | Guzman et al. 1995 |
| pMPV1 | pBAD33_zapC_his_, Cm_r_ | This work |
| pPNV40 | pTrc99A_ftsA^+^, contains P_Trc_ promoter, Amp_r_ | Laboratory collection |
| pPZV33 | pTrc99A_ftsA*, contains P_Trc_ promoter, Amp_r_ | Laboratory collection |

Guzman LM, Belin D, Carson MJ, Beckwith J. Tight regulation, modulation, and high-level expression by vectors containing the arabinose PBAD promoter. J Bacteriol. 1995;**177**:4121-30.
