# Supplementary material for: The hypermorph FtsA* protein has an in vivo role in relieving the Escherichia coli proto-ring block caused by excess ZapC+
Source: PLoS One. 2017 Sep 6;12(9):e0184184. doi: 10.1371/journal.pone.0184184 (PMC5587298; doi:10.1371/journal.pone.0184184)
Supplement: S3 Table — (DOCX) [file pone.0184184.s003.docx]

**S3 Table. Antibodies used in this study.**

| **Antibody** | **Dilution** | **Experiment** | **Source or reference** |
| --- | --- | --- | --- |
| Anti-polyhistidine monoclonal | 1:10000 | Inmunoblotting | Sigma |
| Alexa 594-conjugated anti-rabbit | 1:500 | Immunofluorescence | Invitrogen |
| MVC2 (FtsZ) | 1:400 | Immunofluorescence | Laboratory stock |
| MVC2 (FtsZ) | 1:20000 | Inmunoblotting | Laboratory stock |
| MVC3 (FtsA) | 1:200 | Immunofluorescence | Laboratory stock |
| MVC3 (FtsA) | 1:400 | Inmunoblotting | Laboratory stock |
| MVC1 (ZipA) | 1:500 | Immunofluorescence | Laboratory stock |
| MVC1 (ZipA) | 1:10000 | Inmunoblotting | Laboratory stock |
| MVC9 (FtsQ) | 1:100 | Immunofluorescence | Laboratory stock |
| MVC6 (FtsK) | 1:200 | Immunofluorescence | Laboratory stock |
| MVG1 (FtsN) | 1:500 | Immunofluorescence | Laboratory stock |
| Protein A (peroxidase conjugate) | 1:3000 | Inmunoblotting | Bio Rad |
