# Supplementary figures and images for: The hypermorph FtsA* protein has an in vivo role in relieving the Escherichia coli proto-ring block caused by excess ZapC+
Source: PLoS One. 2017 Sep 6;12(9):e0184184. doi: 10.1371/journal.pone.0184184 (PMC5587298; doi:10.1371/journal.pone.0184184)

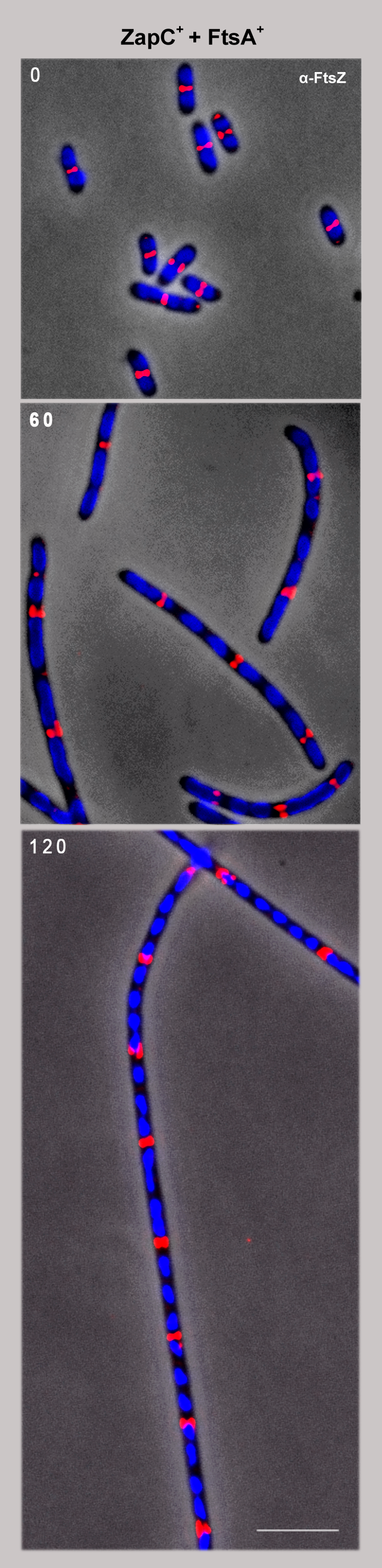

Supplement: S1 Fig — Samples from the cultures overexpressing together zapC+ and ftsA+ were withdrawn at indicated times. Merged images show FtsZ protein visualized using anti-FtsZ and Alexa 594-conjugated anti-rabbit antibody (red signal) and nucleoids visualized using DAPI staining (blue signal). Bar: 5 μm. (TIF) [file pone.0184184.s004.tif]
